# Bisphosphonates induce the osteogenic gene expression in co-cultured human endothelial and mesenchymal stem cells

**DOI:** 10.1111/jcmm.12154

**Published:** 2013-10-31

**Authors:** Viviana Ribeiro, Mónica Garcia, Raquel Oliveira, Pedro S Gomes, Bruno Colaço, Maria Helena Fernandes

**Affiliations:** a CECAV Departamento de Zootecnia, Universidade de Trás-os-Montes e Alto DouroVila Real, Portugal; b FMDUP Laboratory for Bone Metabolism and Regeneration Faculty of Dental Medicine, University of PortoPorto, Portugal

**Keywords:** Alendronate, Zoledronate, osteogenic gene expression, endothelial gene expression, human dermal microvascular endothelial cells (HDMEC), human mesenchymal stem cells (HMSC), co-cultured cells

## Abstract

Bisphosphonates (BPs) are known to affect bone homeostasis and also to have anti-angiogenic properties. Because of the intimate relationship between angiogenesis and osteogenesis, this study analysed the effects of Alendronate (AL) and Zoledronate (ZL) in the expression of endothelial and osteogenic genes on interacting endothelial and mesenchymal stem cells, an issue that was not previously addressed. Alendronate and ZL, 10^−12^–10^−6^ M, were evaluated in a direct co-culture system of human dermal microvascular endothelial cells (HDMEC) and human bone marrow mesenchymal stem cells (HMSC), over a period of 14 days. Experiments with the respective monocultures were run in parallel. Alendronate and ZL caused an initial dose-dependent stimulation in the cell proliferation in the monocultures and co-cultures, and did not interfere with their cellular organization. In HDMEC monocultures, the expression of the endothelial genes CD31, VE-cadherin and VEGFR2 was down-regulated by AL and ZL. In HMSC monocultures, the BPs inhibited VEGF expression, but up-regulated the expression of the osteogenic genes alkaline phosphatase (ALP), bone morphogenic protein-2 (BMP-2) and osteocalcin (OC) and, to a greater extent, osteoprotegerin (OPG), a negative regulator of the osteoclastic differentiation, and increased ALP activity. In co-cultured HDMEC/HMSC, AL and ZL decreased the expression of endothelial genes but elicited an earlier and sustained overexpression of ALP, BMP-2, OC and OPG, compared with the monocultured cells; they also induced ALP activity. This study showed for the first time that AL and ZL greatly induced the osteogenic gene expression on interacting endothelial and mesenchymal stem cells.

## Introduction

Bisphosphonates (BPs) are synthetic analogues of pyrophosphates, which are physiological inorganic molecules regulators of bone mineralization. Bisphosphonates are formed by two phosphate groups covalently linked to a central carbon atom (P-C-P), making it a stable molecule, not hydrolysable, and with a high affinity to the calcium ions on bone matrix surface 1–2. The nitrogen-containing BPs, pamidronate, Alendronate (AL), ibandronate, risedronate and Zoledronate (ZL), are internalized by osteoclasts and repress farnesyl diphosphate synthase, an enzyme of the mevalonate pathway, blocking the prenylation of GTPases (Ras, Rho, Rab) that accumulate in the cytoplasm of osteoclastic cells, reducing their activity 1–2. They act as antiresorptive agents of bone matrix, affecting the metabolism and function of osteoclastic cells and preventing the dissolution of hydroxyapatite crystals 1–2. Bisphosphonates are used to treat bone diseases associated with increased bone resorption caused by a higher osteoclastic activity, such as osteoporosis, Paget’s disease and malignant diseases like multiple myeloma or metastasis to the bone 1–2.

In addition to the relevant effects of BPs in osteoclastic cells and bone resorption, these molecules may also modulate osteoblastic cell behaviour 3 and bone formation 4–5. *In vitro* studies have documented that, at low concentrations, BPs elicited positive effects in the proliferation, differentiation and activity of osteoblastic lineage cells 3–11. In line with this, several studies addressed the incorporation of BPs in bone biomaterials aiming to improve bone formation events and speed up the regeneration process. Thus, inductive effects were observed on osteoblastic cells cultured over these materials 12,13 and also on bone formation following their implantation in animal models of bone regeneration and fracture healing 15–16, including in the presence of metabolic systemic diseases, as in the osteoporotic environment 17–20.

Bisphosphonates are also known to have anti-angiogenic effects, which partly account for their antitumour activity 2–21, and some of the adverse effects, as the avascular osteonecrosis process in areas of high vascularization and bone turnover, such as in the osteonecrosis of the jaw 22–23. *In vitro*, BPs interfere with the functional activity of endothelial cells, namely progenitor cells 24–25, umbilical vein endothelial cells (HUVECs) 25–26, dermal microvascular endothelial cells (HDMECs) 27 and endothelial cells from patients with multiple myeloma 28.

In the bone microenvironment, angiogenesis and osteogenesis are intimately associated, and there is a reciprocal regulation and functional relationship between endothelial cells and osteoblasts during osteogenesis 29. Communication strategies between the two cell types involve cell-to-cell contact at gap junctions 29,30 and a multiplicity of paracrine mechanisms. Thus, endothelial cells secrete a variety of regulatory molecules with a major role in controlling the differentiation and activity of osteoblastic cells, which, in turn, also influence endothelial cells activity through the release of angiogenic growth factors during osteogenesis. This is supported by a variety of *in vitro* studies addressing the interaction of endothelial and osteoblastic cells in different co-culture systems and experimental protocols 30–35, with some in a context of bone regeneration strategies 30–38. These studies have documented that the direct cell-to-cell contact is associated with a reciprocal induction of both phenotypes. Despite this intimate relationship, and the known effects of BPs in the bone metabolism, the influence of these molecules on interacting endothelial and osteoblastic cells has not yet been reported. Considering this, this study analysed the dose- and time-dependent effects of AL and ZL, two widely used BPs 1–2, in a direct co-culture system of human dermal microvascular endothelial cells (HDMEC) and human bone marrow mesenchymal stem cells (HMSC). Cell response was evaluated for cell proliferation, cell morphology and pattern of cell growth. To elucidate subjacent molecular mechanisms, HDMEC/HMSC co-cultures were submitted to fluorescence-activated cell sorting (FACS) for the separation of the two cell populations, and the sorted populations were assessed for the expression of endothelial and osteogenic genes.

## Materials and methods

### Cell cultures

#### Human dermal microvascular endothelial cells

Human dermal microvascular endothelial cells (HDMEC, Sciencell), according to the supplier, were found to stain positive for von Willebrand factor (vWF)/Factor VIII, CD3 and to uptake labelled acetylated low density lipoprotein (DiI-Ac-LDL) – characteristic markers of the endothelial phenotype. Cells were cultured in endothelial cell culture basal medium (EC medium, Sciencell) containing 5% foetal bovine serum (FBS, Sciencell), Penicillin (10 units/ml)/Streptomycin (10 μg/ml) (P/S solution, Sciencell) and a cocktail of endothelial cell growth supplements (ECGS, Sciencell). Incubation was carried out in a humidified atmosphere of 95% air and 5% CO_2_ at 37°C.

#### Human mesenchymal stem cells-bone marrow derived

Human mesenchymal stem cells (HMSC-bm, Innoprot), according to the supplier, were found to stain positive for CD44 and CD90 – characteristic markers of the population phenotype. Cells were cultured in minimum essential medium Eagle, alpha modification (α-MEM, Sigma-Aldrich, Sintra, Portugal) containing 10% FBS (Sigma-Aldrich), Penicillin (10 units/ml)/Streptomycin (10 μg/ml) (P/S solution, Sciencell). Incubation was carried out in a humidified atmosphere of 95% air and 5% CO_2_ at 37°C.

#### Co-culture of HDMEC/HMSC

Human dermal microvascular endothelial cells and HMSC, arising from the third subculture, were co-cultured at a cell density of 2 × 10^4^ cells/cm^2^ HDMEC and 0.5 × 10^4^ cells/cm^2^ HMSC (total cell density, 2.5 × 10^4^ cells/cm^2^). The medium was a mixture (50:50) of EC culture medium and HMSC culture medium. Monocultures of HDMEC and HMSC were used as control; they were seeded at 2.5 × 10^4^ cells/cm^2^ and were maintained in the same experimental conditions as the co-cultures. This protocol was based on our previous work 35 and in the available literature. There is evidence that a higher initial cell density of endothelial cells should be used because of their relatively low growth rate, and the tendency for HMSC to overgrow in the rich medium required for the survival of endothelial cells 37. In addition, we used the same number of plated cells in monocultures and co-cultures (2.5 × 10^4^ cells/cm^2^) to have similar cell-to-cell interaction.

#### Exposure to AL and ZL

Monocultures and co-cultures of HDMEC and HMSC, established as described above, were cultured for 24 hrs. Subsequently, the culture medium was removed and replaced by one containing AL (Sigma-Aldrich®) or ZL (Aclasta®, 5 mg/ml, Novartis Pharma®, Sintra, Portugal) at 10^−12^, 10^−10^, 10^−8^ and 10^−6^ M. Cultures were continued for 14 days, with renewal of the medium containing the tested BPs on days 3, 7 and 10. Control monocultures and co-cultures (absence of AL and ZL) were performed in parallel. The tested BPs concentration range was based on the information reported in the literature regarding cell culture studies, and on preliminary experiments which enable us to exclude the levels that caused rapid cell death. Cell cultures were characterized as follows.

### DNA content

Cell proliferation was estimated by the DNA content, at days 2, 7 and 14. DNA content was analysed by the PicoGreen DNA quantification assay (Quant-iT™ PicoGreen® dsDNA Assay Kit, Molecular Probes Inc., Eugene, OR, USA), according to manufacturer′s instructions. Cultures were treated with Triton X-100 (0.1%; Sigma-Aldrich) and fluorescence was measured on a Elisa reader (Synergy HT, Biotek, Friedrichshall, Germany) at wavelengths of 480 and 520 nm, excitation and emission respectively, and corrected for fluorescence of reagent blanks. The amount of DNA was calculated by extrapolating a standard curve obtained by running the assay with the given DNA standard.

### Immunostaining of F-actin cytoskeleton, CD31 and nucleus

Cultures were fixed in 4% formaldehyde (methanol free, Sigma-Aldrich) for 15 min., permeabilized with 0.1% triton for 5 min., and then incubated in 1% bovine serum albumin (BSA)/PBS for 1 hr.

Human dermal microvascular endothelial cells monocultures and HDMEC/HMSC co-cultures were stained for CD31, with primary CD31 antibody (PECAM-1 (P2B1) mouse anti-human sc-20071; Santa Cruz Biotechnology, Heidelberg, Germany) diluted 1:100 in 1% BSA/PBS (45 min.), then labelled with the secondary antibody [Alexa Fluor 488 goat antimouse IgG1 (ϒ1); Molecular Probes] diluted 1:1000 in 1% BSA/PBS (45 min.); nuclei were stained with 10 μg/ml propidium iodide (Sigma-Aldrich; 10 min.).

Human mesenchymal stem cells monocultures were stained for F-actin, with Alexa Fluor-conjugated phalloidin (Alexa Fluor® 488 Phalloidin: Molecular Probes) diluted 1:100 in 1% BSA/PBS (60 min.). Nuclei were stained with 10 μg/ml propidium iodide (Sigma-Aldrich) diluted in PBS (10 min.).

Stained samples were washed in PBS and covered with Vectashield (Vector Laboratories, Peterborough, UK). Cultures were observed, at days 7 and 14, by Confocal Laser Scanning Microscopy (CLSM) for the cell morphology and cell growth pattern, on a Leica TCP SP2 AOBS (Leica, Werzlar, Germany).

### Fluorescence-activated cell sorting

Human dermal microvascular endothelial cells/HMSC co-cultures were submitted to FACS for the separation of the two cell populations.

Human dermal microvascular endothelial cells and HMSC monocultures and co-cultures were treated with trypsin to detach the cells, at days 7 and 14. Single cells were suspended at a density of 10^5^–10^7^ cells/ml and stained with FITC-conjugated anti-human CD31 antibody (PECAM-1, BD Biosciences, Madrid, Spain), and then washed and re-suspended in a final volume of 250 μl. Using the equipment BD FACSAria™ II system, once the cell population to be sorted has been identified, CD31-positive (labelled cells) and CD31-negative populations were gated and sorted into different collection tubes. Human dermal microvascular endothelial cells and HMSC monocultures were used as control, to confirm CD31 expression or not, and to evaluate the auto-fluorescence. Human dermal microvascular endothelial cells population from the co-culture (cHDMEC) was gated in the CD31 positive cell quadrant, sorted in the exclusion mode and collected into sorting tubes. Human mesenchymal stem cells population from the co-culture (cHMSC) was gated in the CD31 negative cell quadrant, also sorted in the exclusion mode and collected into different sorting tubes. The number of sorted events was 50,000 for each sample. Data processing was performed with FlowJo software 8.7.

### Gene expression by RT-PCR

Control cultures and cultures treated with 10^−12^ and 10^−6^ M AL or ZL were assessed for gene expression by RT-PCR, at days 7 and 14. Analysis was done on HDMEC and HMSC monocultures and, also, on cHDMEC and cHMSC (the populations sorted from the co-culture by FACS). Human dermal microvascular endothelial cells and cHDMEC were evaluated for the housekeeping gene β-actin and for the endothelial genes CD31, vWF, VE-Cadherin and VEGFR2. Human mesenchymal stem cells and cHMSC were assessed for β-actin and for the osteoblastic genes ALP (alkaline phosphatase), BMP-2 (bone morphogenic protein-2), OC (osteocalcin), VEGF-165 and OPG (osteoprotegerin). Total RNA was extracted using the NucleoSpin® RNA II Kit (Macherey-Nagel, Duren, Germnay) according to the manufacturer′s instructions. The concentration and purity of total RNA in each sample were assessed by UV spectrophotometry at 260 nm and by calculating the A260 nm/A280 nm ratio, respectively. Half microgram of RNA was reverse transcribed and amplified (30 cycles) with the Titan One Tube RT-PCR system (Roche® Applied Science, Mannheim, Germany), with an annealing temperature of 55°C. The primers used are listed on Table 1. The PCR products were electrophoresed in a 1% agarose gel, stained with ethidium bromide and semi-quantitatively assessed by densitometry with Image J® software (National Institutes of Health, Bethesda, MD, USA). Data were expressed as normalized ratios by comparing the integrated density values for all tested genes with those for β-actin.

**Table 1:** Primers used on RT-PCR analysis

Gene	5′ primer	3′ primer
vWF	5′-GTTGTGGGAGATGTTTGCCT-3′	5′-TGGAGTACATGGCTTTGCTG-3
CD31	5′-ATG AAG AGC CTG CCG GAC TG-3′	5′-TTC CGT CAC GGT GAC CAG TT-3′
VE-Cad	5′-GAG TCG CAA GAA TGC CAA GT-3′	5′-TAC TTG GTC ATC CGG TTCTG-3′
VEGFR2	5′-TTTGGTTCTGTCTTCCAAAGT-3′	5′-ATGCTCAGCAGGATGGCAA-3′
ALP	5′-ACGTGGCTAAGAATGTCATC-3′	5′-CTGGTAGGCGATGTCCTTA-3′
BMP-2	5′-TCA AGC CAA ACA CAA CAC GC-3′	5′-AGC CAC AAT CCA GTC ATT CC-3′
OPG	5′-AAG GAG CTG CAG TAG GTC AA-3′	5′-CTG CTC GAA GGT GAG GTT AG-3′
OC	5′-CACTCCTCGCCCTATTG-3′	5′-CCC ACA GAT TCC TCT TCT-3′
VEGF165	5′-GAACTTTCTGCTGTCTTG-3′	5′-TTCTTGTCTTGCTCTATCT-3′
β-actin	5′-TGA AGT GTG ACG TGG ACA TC-3′	5′-GGAGGAGCAATGATCTTGAT-3′

### Alkaline phosphatase activity

Human mesenchymal stem cell monocultures and HMSC sorted from the co-culture (cHMSC) were assessed for ALP activity, at days 7 and 14. Analysis was done on control cultures and cultures treated with 10^−12^ and 10^−6^ M AL or ZL. Alkaline phosphatase activity was evaluated in cell lysates (0.1% Triton X-100, 5 min.) by the hydrolysis of *p*-nitrophenyl phosphate in alkaline buffer solution (pH ∼10.3; 30 min., 37°C) and colorimetric determination of the product (*p*-nitrophenol) at 400 nm in an ELISA plate reader (Synergy HT, Biotek). Alkaline phosphatase activity was normalized to total protein content (quantified by Bradford’s method) and was expressed as nmol/min.mg_protein_^−1^. Preliminary experiments showed that ALP activity was not detected in the endothelial cell populations (HDMEC and cHDMEC).

### Statistical analysis

Three independent experiments were performed; in each experiment, five replicas were accomplished for the biochemical assays and three replicas for the qualitative assays. Quantitative results were expressed as the arithmetic mean ± SE. Analysis of results was carried out using IBM SPSS Statistics 19 and statistical analysis was assessed using the one way anova, with a significance level of *P* ≤ 0.05.

## Results

### Cell proliferation, cell morphology and cell growth pattern

**Figure 1:**
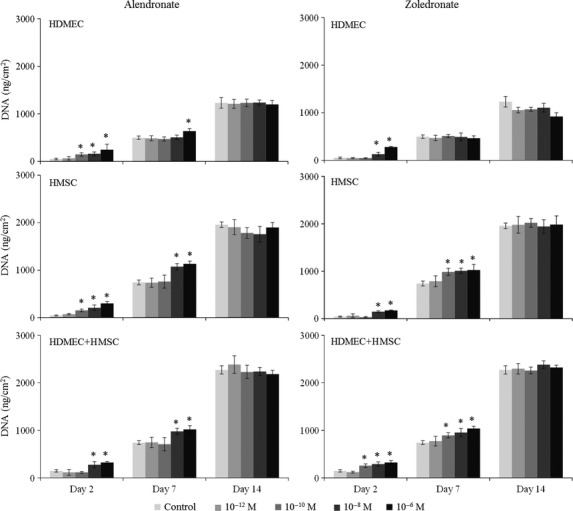
Cell proliferation (DNA content) of monocultured and co-cultured human dermal microvascular endothelial cells and human mesenchymal stem cells in the absence (control) and in the presence of Alendronate (AL) and Zoledronate (ZL), 10^−12^–10^−6^ M. Asterisks (*) significantly different from control (absence of AL or ZL), *P* ≤ 0.05.

Cell proliferation of control HDMEC and HMSC monocultures increased until day 14. Comparatively, HDMEC presented lower values. Co-cultures showed a similar time-dependent pattern; values were comparable to those found in HMSC at days 2 and 7, but higher at day 14. Alendronate increased the proliferation of monocultured and co-cultured HDMEC and HMSC at day 2 (10^−10^–10^−6^ M) and at day 7 (10^−6^ M). Zoledronate induced the proliferation of HDMEC at day 2 (10^−8^ and 10^−6^ M) and that of HMSC at day 2 (10^−8^ and 10^−6^ M) and at day 7 (10^−10^–10^−6^ M); ZL also increased the proliferation of the co-cultured cells, at days 2 and 7 (10^−10^–10^−6^ M). Results are shown in Figure 1.

On CLSM observation, monocultured HDMEC presented rounded morphology, stained intensively for CD31 at the cell boundaries and established perfect cell-to-cell contact. Human mesenchymal stem cells exhibited an elongated morphology, a well-organized F-actin cytoskeleton, cell-to-cell contacts and random growth pattern. On co-cultures, the two populations did not mix up and displayed a characteristic organization, *i.e*. the endothelial cells (CD31-positive cells) formed cord-like structures surrounding the osteoblastic cells. The two cell populations proliferated throughout the culture time, maintaining the same organization. Alendronate and ZL did not cause apparent changes in the organization of the monocultures and co-cultures, at days 7 and 14. However, at day 14, BPs induced some alterations in the cell morphology and organization of the cell layer in HDMEC. Thus, in control cultures, the endothelial cells were organized as a continuous cell layer with tight cell-to-cell junctions whereas, with the BPs, decreased cytoplasmic volume and loss of cell layer integrity appeared evident, particularly with 10^−6^ M BPs. Representative images of the described behaviour are shown as supplementary data (Figs S1–S3).

### Gene expression by RT-PCR

#### HDMEC and cHDMEC populations

**Figure 2:**
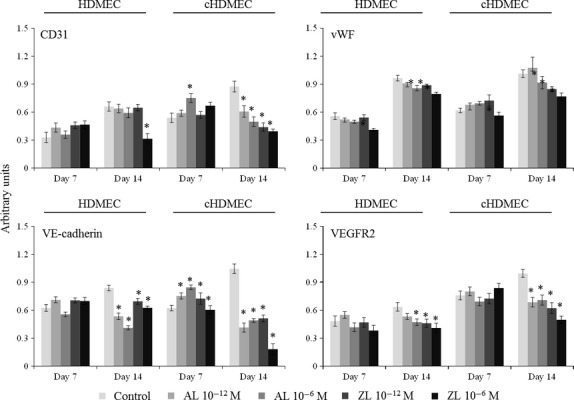
Expression of the endothelial genes CD31, vWF, VE-cadherin and VEGFR2 by human dermal microvascular endothelial cells (HDMEC) monocultures and cHDMEC (the endothelial cell population sorted from the co-culture), in the absence (control) and in the presence of Alendronate (AL) and Zoledronate (ZL), 10^−12^ and 10^−6^ M. Asterisks (*) significantly different from control (absence of AL or ZL), *P* ≤ 0.05.

Control HDMEC and cHDMEC expressed CD31, vWF, VE-cadherin and VEGFR-2. Gene expression increased from day 7 to day 14, and was higher in cHDMEC, Figure 2.

The effects of BPs in the expression of CD31 by HDMC were a slight increase at day 7 (AL 10^−12^ M; ZL 10^−12^ and 10^−6^ M), and a significant decrease following exposure to ZL (10^−6^ M) at day 14. In cHDMEC, the BPs caused an increased expression of CD31 at day 7 (at 10^−6^ M), but an evident dose-dependent inhibition at day 14. Zoledronate had a higher inhibitory effect.

The expression of vWF was reduced, at day 7, in the presence of ZL 10^−6^ M, in both HDMEC and cHDMEC. At day 14, AL and ZL induced an inhibitory effect in both populations, which was greater with ZL 10^−6^ M.

In HDMEC, the BPs elicited a tendency for an increased expression of VE-cadherin at day 7, but a significant decrease at day 14. In cHDMEC, a stimulatory effect was seen at day 7 (AL 10^−12^ and 10^−6^ M; ZL 10^−12^ M) but, at day 14, gene expression was significantly decreased; in the presence of ZL 10^−6^ M, expression of VE-cadherin was barely detected.

The expression of VEGFR2 was reduced in the presence of AL and ZL in both HDMEC and cHDMEC, at day 14, and the inhibitory effect was higher with ZL 10^−6^ M.

#### HMSC and cHMSC populations

**Figure 3:**
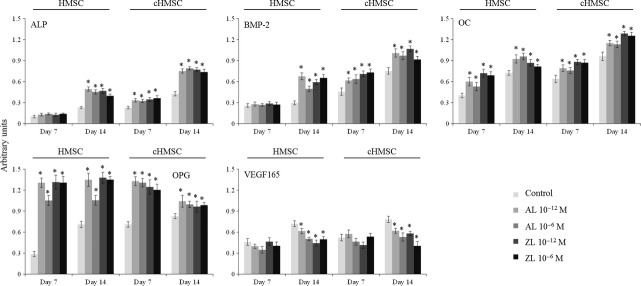
Expression of the osteoblastic genes alkaline phosphatase, BMP-2, OC, OPG and VEGF-165 by human mesenchymal stem cells (HMSC) monocultures and cHMSC (the osteoblastic cell population sorted from the co-culture), in the absence (control) and in the presence of Alendronate (AL) and Zoledronate (ZL), 10^−12^ and 10^−6^ M. Asterisks (*) significantly different from control (absence of AL or ZL), *P* ≤ 0.05.

Human mesenchymal stem cell and cHMSC expressed ALP, BMP-2, OC, OPG and VEGF-165, Figure 3, and gene expression increased from days 7 to 14. However, the population sorted from the co-cultures (cHMSC), exhibited significantly higher gene expression.

The BPs (AL and ZL) increased significantly the expression of ALP, BMP-2, OC and OPG in both HMSC and cHMSC. It is worth to note the high increase in the OPG expression in the presence of AL and ZL; in addition, this effect was higher at day 7. However, AL and ZL caused a decrease in the expression of VEGF-165 in HMSC and cHMSC, particularly at day 14. Zoledronate caused a higher inhibition.

### ALP activity in HMSC and cHMSC populations

**Figure 4:**
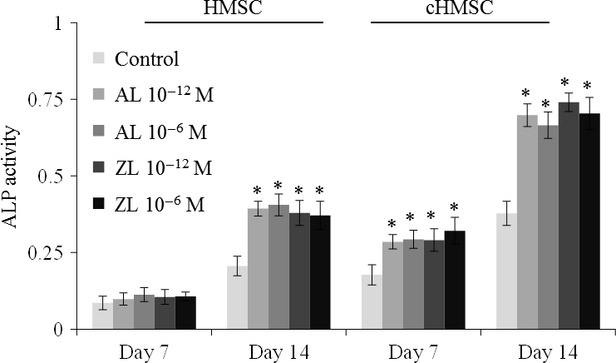
Alkaline phosphatase activity in human mesenchymal stem cells (HMSC) monocultures and cHMSC (the osteoblastic cell population sorted from the co-culture), in the absence (control) and in the presence of Alendronate (AL) and Zoledronate (ZL), 10^−12^ and 10^−6^ M. Asterisks (*) significantly different from control (absence of AL or ZL), *P* ≤ 0.05.

Alkaline phosphatase activity increased from days 7 to 14 in HMSC monocultures and in cHMSC; values were higher in cHMSC. Alendronate and ZL, 10^−12^ and 10^−6^ M, increased significantly ALP activity in monocultured HMSC at day 14, whereas in cHMSC ALP activity was increased at day 7 (∼50%) and at day 14 (∼80%). Results are presented in Figure 4.

## Discussion

In the bone microenvironment, there is a reciprocal interaction between angiogenesis and osteogenesis during physiological and pathological changes in bone turnover 29, and the main objective of this study was to address the dose- and time-dependent effects of AL and ZL in the endothelial and osteogenic gene expression on interacting HDMEC and HMSC.

Alendronate and ZL were tested in a wide concentration range, based on previous *in vitro* studies involving osteoblastic cells 6–11 or endothelial cells 25,26, and also taking into account the difficulty in selecting BPs levels representative to those present in the bone microenvironment under therapeutic dosages. Regarding this, following a 15-min. IV infusion of ZL, a peak plasma concentration of 10^−6^–10^−5^ M has been suggested 39 and, using a bioassay, levels from 0.4 to 5 × 10^−6^ M were reported in saliva and bone specimens retrieved from individuals with BPs-associated osteonecrosis 40. However, because of unique pharmacokinetics of these molecules, levels of BPs that remain in the bone environment are largely unknown. Bisphosphonates have high affinity to bone, preferentially at sites with increased bone remodelling, and can remain locally for a prolonged period of time. They slowly release from bone during osteoclastic resorption and can reattach to adjacent mineral surfaces, being locally recycled 1–2. As recently reviewed 41, the local bone concentration of BPs associated with these dynamics is largely estimated from *in vivo* and *in vitro* studies. The knowledge on BPs distribution in humans is still limited, and there are differences among these molecules in the bone mineral binding affinity 1–2. However, consensual levels are around 10^−12^–10^−9^ M 41, but several reports have suggested that bones with high turnover, like the jaw bones, are more susceptible to deposition of BPs 2–41. Thus, in this study, AL and ZL were tested at 10^−12^–10^−6^ M.

Regarding the co-culture system, a wide variety of experimental conditions have been reported on the co-culture of endothelial and osteoblastic cells, namely regarding cell density, cell ratio, culture medium and culture time 30–38. This is because it is not easy to define the ideal culture conditions to ensure the proliferation and differentiation of two cell populations with distinct growth requirements. In this case, HDMEC is the more sensitive cell type, having higher demands for cell survival and phenotype maintenance 36. With the experimental protocol used in this study, based on a previous work 35, endothelial and osteoblastic phenotypes were achieved and maintained in monoculture and co-culture, appearing as an appropriate model to analyse the effects of BPs in interacting endothelial and osteoblastic cells.

Human dermal microvascular endothelial cells monocultures proliferated throughout the culture time and presented endothelial features, namely the cells with a typical cell cobblestone-like morphology were organized in a continuous cell layer, showed positive CD31 staining at the cell boundaries and expressed the genes for CD31, vWF, VE-cadherin and VEGFR2. Alendronate and ZL caused discrete dose-dependent increase in the cell proliferation at early culture times, and time-dependent effects on gene expression, *i.e*. mixed effects at day 7, but a significant decrease in the expression of CD31, VE-cadherin and VEGFR2 at day 14. Also at day 14, on CLSM images, cells appeared with lower cytoplasmic volume and areas of discontinuity were evident on the cell layer. These morphological alterations might be related to the decreased expression of VE-cadherin, a cell–cell adhesion glycoprotein with a key role in the integrity of intercellular junctions 36. On previous studies, proliferation of HDMEC exposed to ZL was not affected at 10^−6^ and 10^−5^ M, but it was inhibited at higher levels 27, and dose-dependent effects of AL and ZL were reported in endothelial progenitor cells 24–25 and in macrovasculature-derived endothelial cells 25–26. Regarding gene expression, ZL was found to down-regulate the expression of VEGFR2 (at 30 μM) in endothelial cells isolated from the bone marrow of multiple myeloma patients 28. Previous data on the expression of CD31, vWF and VE-cadherin are not available. However, the present results are in line with a variety of studies reporting that, *in vitro*, BPs interfere with the functional activity of endothelial cells and, *in vivo*, they decrease angiogenesis in a variety of experimental and clinical conditions 2–43.

Human mesenchymal stem cell monocultures revealed increased proliferation with culture time, expression of the osteoblastic-related genes ALP, BMP-2, OC, OPG and VEGF-165, and high ALP activity. Alendronate and ZL, caused an initial increase in the cell proliferation, a significant up-regulation in the expression of ALP, BMP-2 and OC and enhanced ALP activity. These observations are consistent with a clear induction of the osteoblastic differentiation of HMSC by the BPs 44. In addition, AL and ZL interfered with the expression of two relevant molecules in the cell–-cell communication involving osteoblasts. They inhibited the expression of VEGF, which is a potent pro-angiogenic factor essential in the formation of functional vessels, and thus with an expected impact in bone formation events 36. However, they caused an early and significant increase in the expression of OPG, a key molecule in the interaction between osteoblasts and osteoclasts, the bone resorbing cells, during bone remodelling. Osteoprotegerin is a decoy receptor that binds to RANKL, an important osteoclastogenic molecule, blocking its binding to the RANK receptor on osteoclasts 45. The observed effects are in agreement with some previous studies addressing a similar concentration range of BPs. Thus, it was shown that AL increased the expression of ALP 8 and OPG 10 in mesenchymal stem cells. Alendronate and ZL also induced the proliferation and expression of BMP-2 in bone marrow stromal cells 7, and ZL induced the expression of BMP-2 and OC 6 and RANKL and OPG 11 in human osteoblastic cells. Regarding the underlying mechanisms, several explanations have been proposed to justify the BPs-mediated increase in cell proliferation of osteogenic-related populations. For instance, Knoch *et al*. 7 reported that ZL, risedronate and AL (10^−8^ M) enhanced the proliferation and promoted the osteoblast differentiation of bone marrow-derived stromal cells collected from different donors. This study showed that, despite donor-to-donor differences on the expression of several osteoblast-specific genes, a sustained increased expression of BMP-2 was found in every experimental situation, in the presence of the BPs 7. Increased BMP-2 expression was also reported in another study with human osteoblastic cells treated with ZL 6. The present study also showed increased BMP-2 expression with AL and ZL. Independently, BPs were found to enhance the proliferation of osteoblastic cells in a process mediate, at least in part, by the activation of the extracellular signal-regulated kinases (ERKs) 46. Taking all together, Erk signalling is known to increase Runx2 stability and transcriptional activity, and the increased expression of BMP-2 may, in addition to the canonical Smad pathway, cooperatively regulate the osteoblastic proliferation and differentiation through a BMP-induced non-Smad Erk signalling pathway 47. Furthermore, anabolic effects of BPs were also found to be related to the stimulation of β-FGF production on osteoblasts 48. Fibroblast growth factors are generally known to play a critical role in bone growth and development, thus stimulating the proliferation of mature osteoblastic and progenitor cells 49–50. Fibroblastic growth factor (FGF) effects were also found to be mediated through the activation of the ERKs pathways 51. Nevertheless, inhibitory effects of BPs in osteoblastic behaviour have also been reported, as recently reviewed 3. As stated by these authors, BPs appear to modulate the behaviour of osteoblastic lineage cells in a dose-dependent manner, *i.e*. they cause increased growth and differentiation at low levels (10^−9^–10^−6^ M) and inhibitory effects at concentrations higher than 10^−5^ M 3.

In the co-cultured HDMEC and HMSC, CLSM observation showed a characteristic organization of the cell layer, with HDMEC forming cord-like structures surrounding HMSC, which is consistent with previous studies showing that the reciprocal interactions between endothelial and osteoblastic cells conditioned the cell distribution 33–35. Also, the endothelial and the osteoblastic genes were overexpressed in the populations sorted from the co-cultures (cHDMEC and cHMSC, respectively), in line with a variety of studies 33–35. In addition to the increased ALP gene expression in cHMSC, activity of this enzyme was also higher in the osteoblastic population sorted from the co-culture, compared to that seen in the monoculture. It is worth to note that regarding the induced osteoblastic gene expression by HMSC co-cultured with endothelial cells as compared to that observed in osteogenic conditions, only few studies addressed this issue, as the vast majority of published works rely on the use of osteogenic-free medium both in co-culture and monoculture environments. Nonetheless, it was reported that the co-culture of endothelial and mesenchymal stem cells results in an increased expression of ALP, up to five times, as compared with the monoculture of MSCs, in the absence or presence of osteogenic inductors, a process that appears to be independent of the activation of Runx2-related pathways 52. In another study, the direct co-culture of bone marrow-derived stromal cells and adult endothelial cells was also found to increase the expression and activity of ALP, despite that no differences were found in an indirect co-culture system of the two cell populations, sustaining the need of direct cell-to-cell contact to achieve this effect 29. Bone morphogenic proteins were also found to be highly expressed in co-culture conditions rather than in monocultured MSCs, in the absence or presence of osteogenic inductors 53; while the expression of VEGF was found to be significantly higher in both endothelial and MSCs in co-culture maintained in osteogenic conditions 54. As described above, the increased expression of ALP has been previously described in several systems of co-cultured endothelial and osteoblastic-related cellular populations 29,30. The subjacent mechanism is supposed to be vastly mediated by the p38 mitogen-activated protein kinase (MAPK) pathway, despite that JNK and Src pathways also seem to play a role in the up-regulation of osteoblastic ALP expression 55. Furthermore, this study has shown that the classical ERK pathway does not play a role in this process 55.

Alendronate and ZL did not interfere with the cell layer organization of the co-cultured HDMEC and HMSC, but they affected significantly the gene expression profile, as assessed in the populations sorted from the co-cultures.

Alendronate and ZL, at day 7, caused a slight stimulation in the expression of CD31, vWF and VE-cadherin by cHDMEC, and increased expression of ALP, BMP-2 and OC by cHMSC, an effect that was not seen in the respective monocultures. These effects suggest that the BPs might favour angiogenesis and osteogenic differentiation in interacting endothelial and osteoblastic cells. However, longer exposure (14 days) inhibited the expression of the endothelial genes by cHDMEC, and the expression of VEGF-165 by cHMCS. This is a relevant effect, as VEGF is essential for blood vessel growth and has been recorded as a potent angiogenic factor by both loss-of-function and gain-of-function studies 56. Within the bone microenvironment, signalling by VEGF seems to promote vascularization during endochondral bone formation, and to regulate the survival and activity of both chondrogenic and osteogenic cells 57. The inhibition of VEGF signalling by blocking its receptor tyrosine kinase, results, *in vivo*, in the regression of the capillaries structure, as verified by the cessation of blood flow and apoptosis of endothelial cells 58. Furthermore, a significant reduction in CD31 immunoreactivity was found, in a time-dependent way 58. The blocking of VEGF signalling was further found to induce capillary regression and reduction in CD31 expression in distinct organs and tissues 59. Accordingly, in this study, the enhanced inhibition of CD31 expression, as well as the inhibition of other endothelial markers, *i.e*., VE-cadherin and VEGFR2, might be related to the decreased expression of VEGF by HMSC, which expression was found to be particularly reduced in established co-cultures grown in the presence of BPs. Nevertheless, in cHMSC, BPs elicited a significant induction in the expression of ALP, BMP-2 and OC, and in the ALP activity, compared with HMSC. In addition, as observed in HMSC monocultures, AL and ZL caused an early and significant increased expression of OPG by cHMSC. This is relevant, as OPG appears to play a role in the effects of PBs in the bone environment. Thus, in a study performed in OPG-deficient mice, as a model for juvenile Paget’s disease, BPs treatment increased the size of the mandibular condyle and normalized growth of the mandibular ramus 60. In addition, in a clinical study involving a group of patients with Paget’s disease, intravenous treatment with pamidronate caused an increase in OPG levels that become statistically significant after 3 and 6 months, and authors suggested that the positive effects of BPs in this disease may be because of either direct or indirect decrease in RANKL-induced bone resorption through increased OPG 61.

As mentioned above, the effects of BPs on interacting endothelial and osteoblastic cells were not previously addressed, and the observed results suggest the possibility of a positive output in bone formation events and increased bone mass, in spite of the apparent deleterious effect in the endothelial cell behaviour. Regarding this, in a recent *in vivo* study, ZL administration increased bone mass in adult mice with no overall structural vascular changes 62. The present *in vitro* observations highlight some of the complex molecular effects of BPs in the bone microenvironment, and appear to be consistent with a variety of studies showing positive effects of BPs in bone formation in models of bone regeneration and fracture healing 3–4. However, as recently reviewed 3,4, the output associated with the complex effects of BPs regarding long-term bone quality and the ability to repair microdamage is largely unknown.

## Conclusion

Monocultured and co-cultured HDMEC and HMSC were exposed to AL or ZL, 10^−12^–10^−6^ M, for 14 days. Alendronate and ZL caused an initial dose-dependent stimulation in the proliferation in the monocultures and co-cultures and no effect at later times. In co-cultures, the cell layer presented a characteristic pattern, with cord-like structures of HDMEC surrounding HMSC, which was not affected by AL and ZL. In HDMEC monocultures, BPs decreased the expression of CD31, VE-cadherin and VEGFR2, whereas, in HMSC monocultures, they inhibited VEGF expression, but caused an overexpression of ALP, BMP-2, OC and, particularly, OPG, and increased ALP activity. In co-cultured endothelial and osteoblastic cells, AL and ZL decreased the expression of endothelial genes, and elicited an earlier and sustained overexpression of ALP, BMP-2 and OC and increased ALP activity. The significant induction of OPG expression was also maintained in the co-cultures. The more relevant observation in this study is the induced osteogenic gene expression in co-cultured HMSC, compared to that on monocultured HMSC, which was observed for the first time in interacting endothelial and osteoblastic cells.

## Supporting information

Figure S1 Representative CLSM images of HDMEC and HMSC monocultures and co-cultures, at control conditions (absence of BPs), day 7. HDMEC and co-cultures were stained for CD31 (green) and nucleus (red); HMSC cultures were stained for F-actin (green) and nucleus (red). HDMEC cultures exhibited a typical cobblestone-like morphology and stained intensively for CD31 at the cell boundaries, establishing perfect cell-to-cell contact. HMSC showed an elongated morphology, a well-organized F-actin cytoskeleton, cell-to-cell contacts and random growth pattern. Co-cultures revealed a characteristic organization, with HDMEC forming cord-like structures surrounding HMSC. Scale bars: 100 μm, upper panels; 25 μm, lower panels.

Figure S2 Representative CLSM images of HDMEC/HMSC co-cultures in the absence (control) and in the presence of Alendronate (AL) and Zoledronate (ZL), 10^-6^ M, days 7 and 14. Cultures treated with AL or ZL, 10^-12^ M, presented similar appearance (data not shown). Co-cultures were stained for CD31 (green) and nucleus (red). Co-cultures exhibited a characteristic organization, with HDMEC forming cord-like structures surrounding HMSC, which was not affected by AL or ZL. Scale bar: 100 μm.

Figure S3 Representative CLSM images of HDMEC monocultures, at control conditions (absence of BPs) and in the presence of Alendronate (AL) and Zoledronate (ZL), 10^-6^ M, day 14. HDMEC were stained for CD31 (green) and nucleus (red). In control cultures, the endothelial cells were organized as a continuous cell layer with tight cell-to-cell junctions whereas, with the BPs, loss of this integrity was evident by the presence of cellular discontinuity in some areas. Scale bar: 25 μm.

## References

[b1] Russell R, Watts NB, Ebetino FH (2008). Mechanisms of action of bisphosphonates: similarities and differences and their potential influence on clinical efficacy. Osteoporos Int.

[b2] Russell RG (2011). Bisphosphonates: the first 40 years. Bone.

[b3] Bellido T, Plotkin LI (2011). Novel actions of bisphosphonates in bone: preservation of osteoblast and osteocyte viability. Bone.

[b4] Allen MR, Burr DB (2011). Bisphosphonate effects on bone turnover, microdamage, and mechanical properties: what we think we know and what we know that we don’t know. Bone.

[b5] Willkinson MJ, Little GL (2011). Bisphosphonates in orthopedic applications. Bone.

[b6] Pan B, To LB, Farrugia AN (2004). The nitrogen-containing bisphosphonate, zoledronic acid, increases mineralisation of human bone-derived cells *in vitro*. Bone.

[b7] Knoch F, Jaquiery C, Kowalsky M (2005). Effects of bisphosphonates on proliferation and osteoblast differentiation of human bone marrow stromal cells. Biomaterials.

[b8] Duque G, Rivas D (2007). Alendronate has an anabolic effect on bone through the differentiation of mesenchymal stem cells. J Bone Miner Res.

[b9] Koch FP, Merkel C, Al-Nawas B (2011). Zoledronate, ibandronate and clodronate enhance osteoblast differentiation in a dose dependent manner - A quantitative *in vitro* gene expression analysis of Dlx5, Runx2, OCN, MSX1 and MSX2. J Craniomaxillofac Surg.

[b10] Ohe J-Y, Kwon Y-D, Lee H-W (2012). Bisphosphonates modulate the expression of OPG and M-CSF in hMSC-derived osteoblasts. Clin Oral Invest.

[b11] Koch FP, Merkel C, Ziebart T (2012). Influence of bisphosphonates on the osteoblast RANKL and OPG gene expression *in vitro*. Clin Oral Invest.

[b12] Greiner S, Kadow-Romacker A, Lubberstedt M (2007). The effect of zoledronic acid incorporated in a poly(D, L-lactide) implant coating on osteoblasts *in vitro*. J Biomed Mater Res A.

[b13] Boanini E, Torricelli P, Gazzano M (2008). Alendronate-hydroxyapatite nanocomposites and their interaction with osteoclasts and osteoblast-like cells. Biomaterials.

[b14] Panzavolta S, Torricelli P, Bracci B (2009). Alendronate and Pamidronate calcium phosphate bone cements: setting properties and *in vitro* response of osteoblast and osteoclast cells. J Inorg Biochem.

[b15] Tatli U, Stün YU, Kürkçü M (2011). Effects of zoledronic acid on healing of mandibular fractures: an experimental study in rabbits. J Oral Maxillofac Surg.

[b16] Agholme F, Andersson T, Tengvall P (2012). Local bisphosphonate release versus hydroxyapatite coatings for stainless stell screw fixation in rat tibiae. J Mat Sci.

[b17] Pampu AA, Dolanmaz D, Tuz HH (2006). Experimental evaluation of the effects of zoledronic acid on regenerate bone formation and osteoporosis in mandibular distraction osteogenesis. J Oral Maxillofac Surg.

[b18] Stadelmann VA, Gauthier O, Terrier A (2008). Implants delivering bisphosphonate locally increase periprosthetic bone density in an osteoporotic sheep model. A pilot study. Eur Cell Mater.

[b19] Verron E, Gauthier O, Janvier P (2010). *In vivo* bone augmentation in an osteoporotic environment using bisphosphonate-loaded calcium deficient apatite. Biomaterials.

[b20] Qi M, Hu J, Li J (2012). Effect of Zoledronate acid treatment on osseointegration and fixation of implants in autologous iliac bone grafts in ovariectomized rabbits. Bone.

[b21] Clézardin P, Benzaïd I, Croucher PI (2011). Bisphosphonates in preclinical bone oncology. Bone.

[b22] Sarasquete ME, Gonzalez M, San MiguelJF (2009). Bisphosphonate-related osteonecrosis: genetic and acquired risk factors. Oral Dis.

[b23] Yarom N, Yahalom R, Shoshani Y (2007). Osteonecrosis of the jaw induced by orally administered bisphosphonates: incidence, clinical features, predisposing factors and treatment outcome. Osteoporos Int.

[b24] Yamada J, Tsuno NH, Kitayama J (2009). Anti-angiogenic property of zoledronic acid by inhibition of endothelial progenitor cell differentiation. J Surg Res.

[b25] Ziebart T, Pabst A, Klein MO (2011). Bisphosphonates: restrictions for vasculogenesis and angiogenesis: inhibition of cell function of endothelial progenitor cells and mature endothelial cells *in vitro*. Clin Oral Investig.

[b26] Hashimoto K, Morishige K, Sawada K (2007). Alendronate suppresses tumor angiogenesis by inhibiting Rho activation of endothelial cells. Biochem Biophys Res Commun.

[b27] Michailidou M, Brown HK, Lefley DV (2010). Microvascular endothelial cell responses *in vitro* and *in vivo*: modulation by zoledronic acid and paclitaxel?. J Vasc Res.

[b28] Scavelli C, Pietro G, Cirulli T (2007). Zoledronic acid affects over-angiogenic phenotype of endothelial cells in patients with multiple myeloma. Mol Cancer Ther.

[b29] Villars F, Bordenave L, Bareille R (2000). Effect of human endothelial cells on human bone marrow stromal cell phenotype: role of VEGF?. J Cell Biochem.

[b30] Villars F, Guillotin B, Amedee T (2002). Effect of HUVEC on human osteoprogenitor cell differentiation needs heterotypic gap junction communication. Am J Physiol Cell Physiol.

[b31] Stahl A, Wenger A, Webe H (2004). Bi-directional cell contact-dependent regulation of gene expression between endothelial cells and osteoblasts in a three-dimensional spheroidal coculture model. Biochem Biophys Res Commun.

[b32] Guillotin B, Bareille R, Bourget C (2008). Interaction between human umbilical vein endothelial cells and human osteoprogenitors triggers pleitropic effect that may support osteoblastic function. Bone.

[b33] Aguirre A, Planell JA, Engel E (2010). Dynamics of bone marrow-derived endothelial progenitor cell/mesenchymal stem cell interaction in co-culture and its implications in angiogenesis. Biochem Biophys Res Commun.

[b34] Saleh F, Whyte M, Genever P (2011). Effects of endothelial cells on human mesenchymal stem cells activity in a three-dimensional *in vitro* model. Eur Cells Mater.

[b35] Laranjeira M, Fernandes MH, Monteiro FJ (2012). Reciprocal induction of human dermal microvascular endothelial cells and human mesenchymal stem cells: time-dependent profile in a co-culture system. Cell Prolif.

[b36] Kanczler JM, Oreffo ROC (2008). Osteogenesis and angiogenesis: the potential for engineering bone. Eur Cells Mater.

[b37] Unger RE, Sartoris A, Peters K (2007). Tissue-like self-assembly in cocultures of endothelial cells and osteoblasts and the formation of microcapillary-like structures on three-dimensional porous biomaterials. Biomaterials.

[b38] Hofmann A, Ritz U, Verrier S (2008). The effect of human osteoblasts on proliferation and neo-vessel formation of human umbilical vein endothelial cells in a long-term 3D co-culture on polyurethane scaffolds. Biomaterials.

[b39] Skerjanec A, Berenson J, Hsu C (2003). The pharmacokinetics and pharmacodynamics of zoledronic acid in cancer patients with varying degrees of renal function. J Clin Pharmacol.

[b40] Scheper MA, Badros A, Salama AR (2009). A novel bioassay model to determine clinically significant bisphosphonate levels. Support Care Cancer.

[b41] Cremers S, Papapoulos S (2011). Pharmacology of bisphosphonates. Bone.

[b42] Yamashita J, Koi K, Yang DY (2011). Effect of Zoledronate on oral wound healing in rats. Clin Cancer Res.

[b43] Tsai S-H, Huang P-H, Chang W-C (2012). Zoledronate inhibits ischemia-induced neovascularization by impairing the mobilization and function of endothelial progenitor cells. PLoS ONE.

[b44] Aubin J, Bilezikian JP, Raisz LG, Martin TJ (2008). Mesenchymal stem cell and osteoblast differentiation. Principles of bone biology.

[b45] Boyce BF, Xing L (2007). Biology of RANK, RANKL, and osteoprotegerin. Arthritis Res Ther.

[b46] Mathov I, Plotkin LI, Sgarlata CL (2001). Extracellular signal-regulated kinases and calcium channels are involved in the proliferative effect of bisphosphonates on osteoblastic cells *in vitro*. J Bone Miner Res.

[b47] Jun JH, Yoon W-J, Seo S-B (2010). BMP2-activated Erk/MAP kinase stabilizes Runx2 by increasing p300 levels and histone acetyltransferase activity. J Biol Chem.

[b48] Giuliani N, Girasole G, Pedrazzoni M (1995). Alendronate stimulates β-FGF production and mineralized nodule formation in human osteoblastic cells and osteoblastogenesis in human bone marrow cultures. J Bone Miner Res.

[b49] Kotev-Emeth S, Savion N, Pri-chen S (2000). Effect of maturation on the osteogenic response of cultured stromal bone marrow cells to basic fibroblast growth factor. Bone.

[b50] Hughes-Fulford M, Li CF (2011). The role of FGF-2 and BMP-2 in regulation of gene induction, cell proliferation and mineralization. J Orthop Surg Res.

[b51] Raucci A, Bellosta P, Grassi R (2008). Osteoblast proliferation or differentiation is regulated by relative strengths of opposing signaling pathways. J Cell Physiol.

[b52] Xue Y, Xing Z, Hellem S (2009). Endothelial cells influence the osteogenic potential of bone marrow stromal cells. Biomed Eng Online.

[b53] Xue Y, Xing Z, Bolstad AI (2013). Co-culture of human bone marrow stromal cells with endothelial cells alters gene expression profiles. Int J Artif Organs.

[b54] Torbjorn TO, Blois AL, Xue Y (2012). Osteogenic stimulatory conditions enhance growth and maturation of endothelial cell microvascular networks in culture with mesenchymal stem cells. J Tissue Eng.

[b55] Sven H, Lampert FM, Orimo H (2009). Up-regulation of alkaline phosphatase expression in human primary osteoblasts by cocultivation with primary endothelial cells is mediated by p38 mitogen–activated protein kinase–dependent mRNA stabilization. Tissue Eng Part A.

[b56] Richard RP, Richardson CD, Sato TN (2002). Orchestration of angiogenesis and arteriovenous contribution by angiopoietins and vascular endothelial growth factor (VEGF). Proc Natl Acad Sci USA.

[b57] Gerber HP, Vu TH, Ryan AM (1999). VEGF couples hypertrophic cartilage remodeling, ossification and angiogenesis during endochondral bone formation. Nat Med.

[b58] Baffert F, Le T, Sennino B (2006). Cellular changes in normal blood capillaries undergoing regression after inhibition of VEGF signaling. Am J Physiol Heart Circ Physiol.

[b59] Kamba T, Tam BY, Hashizume H (2006). VEGF-dependent plasticity of fenestrated capillaries in the normal adult microvasculature. Am J Physiol Heart Circ Physiol.

[b60] Kimura M, Miyazawa K, Tabuchi M (2008). Bisphosphonate treatment increases the size of the mandibular condyle and normalizes growth of the mandibular ramus in osteoprotegerin-deficient mice. Calcif Tissue Int.

[b61] Martini G, Gennari L, Merlotti D (2007). Serum OPG and RANKL levels before and after intravenous bisphosphonate treatment in Paget’s disease of bone. Bone.

[b62] Soki FN, Li X, Berry J (2013). The effects of zoledronic acid in the bone and vasculature support of hematopoietic stem cell niches. J Cell Biochem.

[b63] Roux C (2009). Potential effects of bisphosphonates on bone ultrastructure. Osteoporos Int.

