# Supplementary figures and images for: Bisphosphonates induce the osteogenic gene expression in co-cultured human endothelial and mesenchymal stem cells
Source: J Cell Mol Med. 2013 Oct 31;18(1):27–37. doi: 10.1111/jcmm.12154 (PMC3916115; doi:10.1111/jcmm.12154)

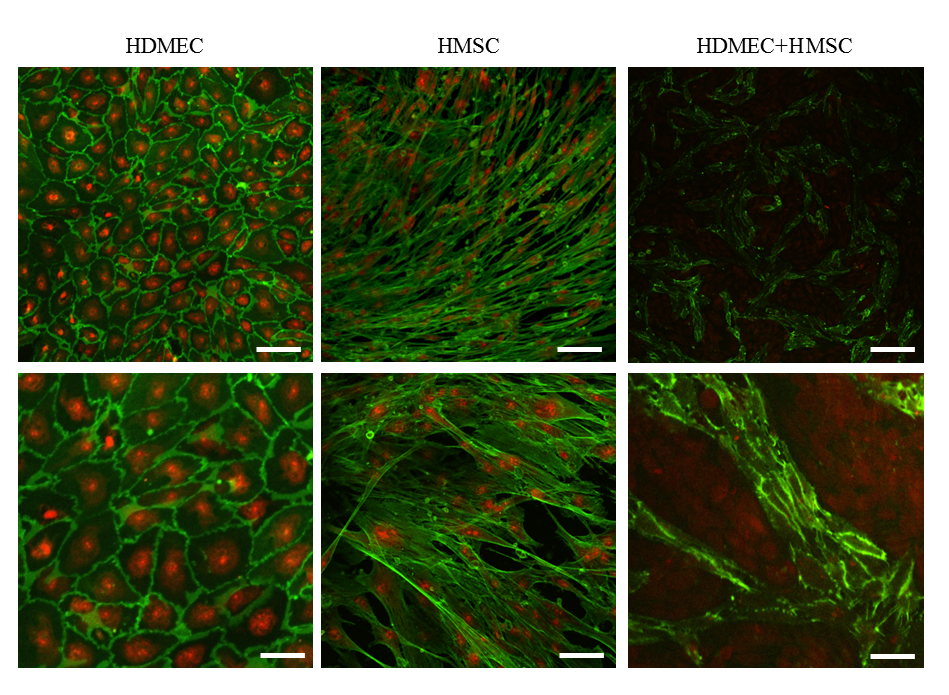

Supplement: Figure S1 — Representative CLSM images of HDMEC and HMSC monocultures and co-cultures, at control conditions (absence of BPs), day 7. HDMEC and co-cultures were stained for CD31 (green) and nucleus (red); HMSC cultures were stained for F-actin (green) and nucleus (red). HDMEC cultures exhibited a typical cobblestone-like morphology and stained intensively for CD31 at the cell boundaries, establishing perfect cell-to-cell contact. HMSC showed an elongated morphology, a well-organized F-actin cytoskeleton, cell-to-cell contacts and random growth pattern. Co-cultures revealed a characteristic organization, with HDMEC forming cord-like structures surrounding HMSC. Scale bars: 100 μm, upper panels; 25 μm, lower panels. [file jcmm0018-0027-sd1.tif]

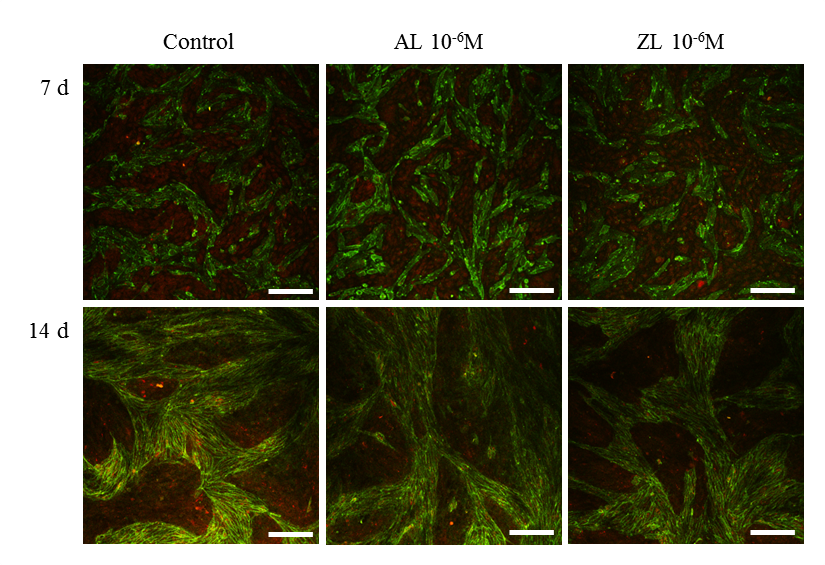

Supplement: Figure S2 — Representative CLSM images of HDMEC/HMSC co-cultures in the absence (control) and in the presence of Alendronate (AL) and Zoledronate (ZL), 10-6 M, days 7 and 14. Cultures treated with AL or ZL, 10-12 M, presented similar appearance (data not shown). Co-cultures were stained for CD31 (green) and nucleus (red). Co-cultures exhibited a characteristic organization, with HDMEC forming cord-like structures surrounding HMSC, which was not affected by AL or ZL. Scale bar: 100 μm. [file jcmm0018-0027-sd2.tif]

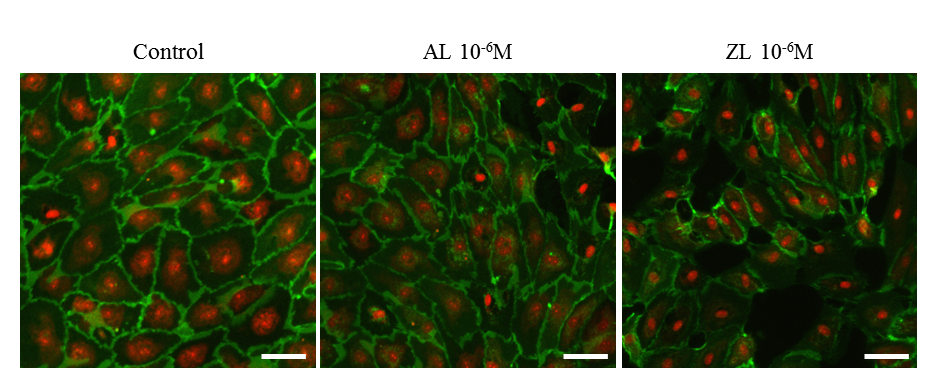

Supplement: Figure S3 — Representative CLSM images of HDMEC monocultures, at control conditions (absence of BPs) and in the presence of Alendronate (AL) and Zoledronate (ZL), 10-6 M, day 14. HDMEC were stained for CD31 (green) and nucleus (red). In control cultures, the endothelial cells were organized as a continuous cell layer with tight cell-to-cell junctions whereas, with the BPs, loss of this integrity was evident by the presence of cellular discontinuity in some areas. Scale bar: 25 μm. [file jcmm0018-0027-sd3.tif]
